# Integrative analysis of metabolomics and transcriptomics to uncover biomarkers in sepsis

**DOI:** 10.1038/s41598-024-59400-0

**Published:** 2024-04-27

**Authors:** Wenhao Chen, Wentao Guo, Yang Li, Muhu Chen

**Affiliations:** Department of Emergency Medicine, The Affiliated Hospital of Southwest Medical University, Luzhou, Sichuan China

**Keywords:** Sepsis, Metabolomics, RNA sequencing, Single-cell RNA sequencing, Biomarkers, DNA, Metabolomics, RNA, Biomarkers, Diseases

## Abstract

To utilize metabolomics in conjunction with RNA sequencing to identify biomarkers in the blood of sepsis patients and discover novel targets for diagnosing and treating sepsis. In January 2019 and December 2020, blood samples were collected from a cohort of 16 patients diagnosed with sepsis and 11 patients diagnosed with systemic inflammatory response syndrome (SIRS). Non-targeted metabolomics analysis was conducted using liquid chromatography coupled with mass spectrometry (LC–MS/MS technology), while gene sequencing was performed using RNA sequencing. Afterward, the metabolite data and sequencing data underwent quality control and difference analysis, with a fold change (FC) greater than or equal to 2 and a false discovery rate (FDR) less than 0.05.Co-analysis was then performed to identify differential factors with consistent expression trends based on the metabolic pathway context; KEGG enrichment analysis was performed on the crossover factors, and Meta-analysis of the targets was performed at the transcriptome level using the public dataset. In the end, a total of five samples of single nucleated cells from peripheral blood (two normal controls, one with systemic inflammatory response syndrome, and two with sepsis) were collected and examined to determine the cellular location of the essential genes using 10× single cell RNA sequencing (scRNA-seq). A total of 485 genes and 1083 metabolites were found to be differentially expressed in the sepsis group compared to the SIRS group. Among these, 40 genes were found to be differentially expressed in both the metabolome and transcriptome. Functional enrichment analysis revealed that these genes were primarily involved in biological processes related to inflammatory response, immune regulation, and amino acid metabolism. Furthermore, a meta-analysis identified four genes, namely ITGAM, CD44, C3AR1, and IL2RG, which were highly expressed in the sepsis group compared to the normal group (*P* < 0.05). Additionally, scRNA-seq analysis revealed that the core genes ITGAM and C3AR1 were predominantly localized within the macrophage lineage. The primary genes ITGAM and C3AR1 exhibit predominant expression in macrophages, which play a significant role in inflammatory and immune responses. Moreover, these genes show elevated expression levels in the plasma of individuals with sepsis, indicating their potential as valuable subjects for further research in sepsis.

## Introduction

The disease sepsis is characterized by life-threatening organ dysfunction caused by an unregulated host response to infection^1^. According to a study conducted in 2020, sepsis affects approximately 480,000 individuals globally, leading to 90,000 fatalities, which accounts for 11.19% of the total number of deaths^2^. The World Health Organization (WHO) designated sepsis as a top concern for global health and called on nations to diminish the worldwide impact of sepsis^3^. At present, the conventional approach to treating sepsis involves source management, prompt resuscitation, timely administration of antibiotics, and therapy to support organ function. Nevertheless, despite numerous improvements in sepsis management approaches in the past few years, the fatality rate of sepsis remains elevated. Because sepsis can present in various ways, diagnosing sepsis remains a difficult task for clinicians, as it lacks speed, sensitivity, and specificity. Consequently, healthcare professionals still encounter significant obstacles when it comes to diagnosing, treating, and managing septic patients^4^. It is crucial to precisely determine sepsis, uncover its intricate molecular characteristics, and develop numerous biomarkers and precise detection methods for prompt identification, early alert, and anticipation of sepsis, enabling patients to obtain precise and efficient interventions like timely diagnosis and treatment.

Metabolomics is an investigative technique that measures the quantities of all metabolites present in a biological system and establishes the correlation between metabolites and physiological as well as pathological alterations^5^. The majority of the analytes consist of small compounds weighing less than 1500 Da. These substances serve as significant markers for physiological or pathological conditions, aiding in the comprehension of disease development and advancement^6^.Various research studies have utilized metabolomic investigations to discover new biomarkers linked to the advancement, mechanisms, and prognosis of sepsis, revealing diverse metabolic profiles in individuals with sepsis^2,7,8^. The investigations conducted in this study exclusively utilized metabolomics methodologies, such as differential analysis and pathway enrichment analysis, to elucidate the metabolic disruptions that underlie the mechanisms of sepsis. The discovery of co-expression patterns using multi-omics data could provide fresh understanding into the mechanisms of sepsis. RNA sequencing, a widely employed technique for gene expression analysis and identification of novel RNAs^9^, has emerged as a highly sophisticated method for transcriptome analysis^10^.Nevertheless, conventional RNA sequencing samples are acquired from heterogeneous cells, and while sequencing enables the identification of differentially expressed genes among multiple cell populations, it does not ascertain the genes accountable for the dissimilarities between cells^11^.The use of single-cell RNA sequencing (scRNA-seq) has had a profound impact on transcriptomics research by providing insights into gene expression at the cellular level^12^, thereby enhancing our understanding of gene localization and expression in distinct cell populations. By combining metabolomics and transcriptome analysis, one can investigate the levels of metabolites and mRNAs, capitalizing on the disparities and synergies between these two histological studies. This approach enables a comprehensive assessment of gene expression, unveiling novel findings that are not attainable through conventional individual histology. Moreover, it facilitates a comprehensive exploration of the mechanisms underlying disease, growth, and organismal development. The advancement of multi-omics detection and analysis technology presents an opportunity for a significant breakthrough in creating a precise detection system for early diagnosis, early warning, and prediction of sepsis. The proposed system will be grounded in evidence-based medicine and will make use of multi-omics big data^13^. Multi-omics analyses have the potential to offer valuable insights into biological functions through mutual validation, which may not be discernible from a single dataset alone.

This study aims to discover new targets for diagnosing and treating sepsis by utilizing metabolomics, transcriptomics, and single-cell sequencing techniques on identical biological samples. The objective is to understand the levels of expression and cellular lineage localization of the primary targets, and establish a foundation for future in vivo functional investigations.

## Materials and methods

### Subject recruitment and blood collection

Included in the study were 16 individuals diagnosed with sepsis and 11 individuals diagnosed with SIRS who were admitted to the EICU at Southwest Medical University Hospital between January 2019 and December 2020.The eligibility requirements included: (1) sepsis patients who were admitted to the EICU; (2) patients who satisfied the Sepsis 3.0 criteria (infection + SOFA score ≥ 2) that were collaboratively published by the American Society of Critical Care Medicine and the European Society of Intensive Care Medicine in 2016; (3) patients aged between 18 and 80 years; and (4) patients who expressed their willingness to take part in the study and completed an informed consent form, or their legal representatives did so. Patients who had experienced organ failure, immune system disorders, or haematological disorders in the past, as well as those who did not want to participate in the trial, were excluded. Additionally, patients with SIRS were considered as other patients who were admitted to the EICU concurrently, such as those who had undergone post-traumatic surgery or other procedures. The trial received approval from the Ethics Committee of the Affiliated Hospital of Southwest Medical University (Ethics No.ky2018029), Clinical Trial Registration No. The study ChiCTR1900021261 adheres to the principles of the Declaration of Helsinki.

### Sequencing of RNA

The process of gene sequencing was carried out with the help of UW Genetics located in Shenzhen, China. Total RNA from peripheral blood cells was extracted with TRIZOL (Invitgen, Carlsbad, CA, USA).first-strand cDNA was synthesised from 800 to 1000 ng of total RNA using the Clontech Smarter PCRcDNA Synthesis Kit.First, CDS primer IIA was used to anneal to the PolyA+ tails of the transcripts, followed by the SMARTScribe™ Reverse Transcriptase for the synthesis of the first-strand cDNA. Next, a Clontech PrimeStar GXL DNA polymerase and 5'-ʹprimer IIA (5'-AAGCAGTGGTATCAACGCAGAGTAC-3') were used to conduct a large-scale polymerase amplification. Finally, a cDNA library of the gene was constructed. The libraries were detected and measured utilizing an Agilent 2100 Bioanalyser (Thermo Fisher Science, Massachusetts, USA) and real-time QPCR (TaqMan probe).The libraries were sequenced by the DNBSEQ platform (Welltec Shenzhen, China).The data was processed and analyzed for differences using the iDEP 1.0^14^ website (http //bioinformatics.sdstate.edu/idep/), applying logarithmic transformation. We then screened for genes that were differentially expressed between the SIRS and sepsis groups, using the criteria of |Fc| (fold change) FDR2.0 and a false discovery rate (FDR) of less than 0.01. Homogeneity in the samples was assessed using box plots, and outlier samples were removed by conducting principal component analysis (PCA) on the two datasets. The sequence data analyzed in this research is available in the Chinese National Genebank database (CNGBdb) and can be accessed at https://db.cngb.org/. The accession number is CNP0002611.

### Non-targeted metabolomics analysis

For this study, the non-targeted metabolomics analyses were conducted using liquid chromatography coupled with mass spectrometry (LC–MS/MS technology). The Q Exactive HF, a high-resolution mass spectrometer from Thermo Fisher Scientific in the USA, was utilized to enhance metabolite coverage. Data acquisition was performed in both positive and negative ion modes.The compound Discoverer 3.1 software from Thermo Fisher Scientific, USA,was utilized for LC–MS/MS data processing, primarily for extracting peaks, aligning peaks,and identifying compounds. The metabolomics R package metaX^15^, online analysis software MetaboAnalyst 5.0 (https//www.metaboanalyst.ca/home.xhtml), and Metabolome Information Analysis Process were utilized for data preprocessing, statistical analysis, metabolite classification annotation, and functional annotation. Multivariate statistical analyses (PCA and OPLS-DA and univariate analyses [multiples of variation (Fold-Change, FC) and t-test)] were used in combination to screen for differential metabolites between groups. PCA and OPLS-DA were applied to model the relationship between metabolite expression and sample category, which led to the prediction of sample category, and the combination of multiplicity of variance (FV) and t-test was used to ultimately identify the differential metabolites between groups. A PCA model was established between the comparative analysis group (two groups) to observe the distribution and separation trend of the two groups of samples. The data is log2 converted before the PCA model is established, and the data is scaled by the Pareto scaling method.

Unlike principal component analysis, partial least squares-discriminant analysis (OPLS-DA) is a supervised statistical method. The difference between classification groups can be reflected to the greatest extent. This method uses partial least squares regression to establish a relationship model between metabolite expression and sample categories to achieve modeling prediction of sample categories. At the same time, the variable importance in projection (VIP) is used to measure the influence intensity and interpretation ability of each metabolite expression pattern on the classification and discrimination of each group of samples, thus assisting in the screening of metabolic markers. It is generally considered that VIP greater than 1 Indicates that this variable has a significant effect on the classification of sample categories.

The OPLS-DA model between the comparative group (two groups) was established after log2-log conversion of the data, and the method used for scaling is Par. The sevenfold cross validation was performed during modelling.To evaluate the model, the OPLS-DA model was subjected to 200 response permutation tests(RPT).

### Combined metabolomics and transcriptomics analysis

Metabolomic co-transcriptional investigations are frequently employed to investigate possible regulatory network mechanisms in organisms, and to gain a comprehensive understanding of the regulatory patterns and mechanisms between individual molecules by examining the expression levels of mRNAs and metabolites.The OMICSHARE platform (https //www.omicshare.com/tools/) was used to submit transcriptomic and metabolomics data for the construction of heatmaps in intergroup correlation analysis. Additionally, the MetaboAnalyst 5.0 platform (https //www.metaboanalyst.ca/ MetaboAnalyst/home.xhtml) was utilized to obtain cross-targets of differential genes and differential metabolites.

### Meta-analysis

In order to further explore the variation in gene expression of crossover genes among different populations at the transcriptional level, we acquired the data from the publicly available GEO database (https//www.ncbi.nlm.nih.gov/geo/).Five peripheral blood samples from sepsis patients were downloaded (GSE6535^16^, GSE12624^17^, GSE28750^18^, GSE63042^19^, GSE74224^20^).The datasets mentioned above were standardized (using log2 logarithmic) into two groups: sepsis group (Sepsis) and SIRS group (NC). A comprehensive Meta-analysis was conducted using R-package, where a MultiMeta20 analysis was performed on individual genes within the same group but from different datasets to confirm the consistency of the expression pattern of the target genes in this research.

### Single-cell RNA sequencing

The blood cells in the periphery consist of various types of cells, and scRNA-seq analysis assists scientists in identifying specific genes within these cells in the tissue. This research examined the cellular lineage localization of specific target genes by utilizing 10× scRNA-seq. The procedures were executed in accordance with the operational guidebook of the organization. A total of five blood samples, including two with NC, one with Systemic Inflammatory Response Syndrome (SIRS), and two with Sepsis, were gathered and combined. Raw data produced through high-throughput sequencing were in the FASTQ format and underwent mass counting using CellRanger (http//support.), the official software of 10× Genomics. The SEURAT software package was used for additional quality control of the data after processing with the latest version of Cell Ranger software from 10× Genomics (https://www.10xgenomics.com/single-cell-gene-expression/software/pipelines/latest/what-is-cell-ranger). Principal component analysis (PCA) was conducted to analyze gene expression data and reduce its linear dimensionality. The resulting PCA outcomes were then visualized in two dimensions using t-distributed Stochastic Neighbor Embedding (tSNE). The FindAllMarkers function was used to identify marker genes, which were then visualized using the VlnPlot and FeaturePlot functions. Using the SingleR^21^ software package, we calculated the correlation between the expression profiles of the cells to be identified and the reference dataset. Then, we assigned the cell types with the strongest correlation in the reference dataset to the cells to be identified. This allowed us to create a single-cell library that is relevant to sepsis. The main objectives examined in the aforementioned studies were entered into the single-cell library in order to determine the cellular localization of the genes of interest.

### Ethical approval and consent to participate

All experimental protocols were approved by the clinical ethics committee of Southwest Medical University. All methods were carried out in accordance with relevant guidelines and regulations. The informed consent was obtained from all subjects and/or their legal guardian(s). The clinical ethics committee of Southwest Medical University (Ethics No: KY2018029) approved this study and the clinical trial registration (ChiCTR1900021261).

### Consent to publication

All the authors agreed to publish.

## Results

### Clinical information

The mean ± standard deviation was calculated for gender, age, ALT, total bilirubin, creatinine, high-sensitivity troponin, BNP, total leukocyte count, neutrophil count, monocyte count, lymphocyte count, prothrombin time, PT-INR, and procalcitoninogen in a group of 16 patients with sepsis and 11 SIRS controls through statistical analysis. Table 1 displays the clinical data. The patients in the SIRS group were a collective of individuals admitted to the EICU following trauma surgery, exhibiting non-infectious factors. The findings indicated that individuals in the sepsis cohort exhibited markedly elevated markers of inflammation and deterioration in organ function.

**Table 1:** Clinical characteristics of all subjects.

Clinical variable	Sepsis group (N = 16)	SIRS group (N = 11)	*P* value
Gender (F/M)	6/10	4/7	#
Age (years)	55.7143 ± 9.4794	47 ± 14.1228	#
ALT	54.0143 ± 46.3081	61.6909 ± 61.4077	0.564
Total bilirubin (Umol/L)	22.6125 ± 18.7188	18.7188 ± 7.8166	0.05
Creatinine (Umol/L)	144.6429 ± 81.0635	94.9455 ± 65.4746	0.357
High-sensitivity troponin (ug/L)	0.1910 ± 0.2877	0.0813 ± 0.1413	0.506
BNP(ng/L)	2116.4714 ± 9929.0081	673.086 ± 1215.9294	0.018
White blood cells (10^9^/L)	13.8 ± 8.4124	11.4082 ± 3.7405	0.001
Neutrophils (10^9^/L)	12.5557 ± 8.0865	9.6809 ± 3.5309	0.006
Monocytes (10^9^/L)	0.4686 ± 1.9073	0.6264 ± 0.2915	0.018
Lymphocytes (10^9^/L)	0.7229 ± 5.3228	1.0227 ± 0.3724	0.012
Thrombin time (s)	15.2143 ± 0.888	16 ± 2.1157	0.001
PT-INR	1.3143 ± 0.1412	1.2055 ± 0.1154	0.05
PCT (ng/mL)	48.5886 ± 37.8257	10.9645 ± 25.4910	0.001

Gender, age, total white blood cell count, neutrophil count, monocyte count, platelet count, PCT and BNP were statistically analysed in 16 septic patients and 11 normal controls and expressed as mean ± standard deviation.

^#^No statistical significance.

### RNA sequencing

**Figure 1:**
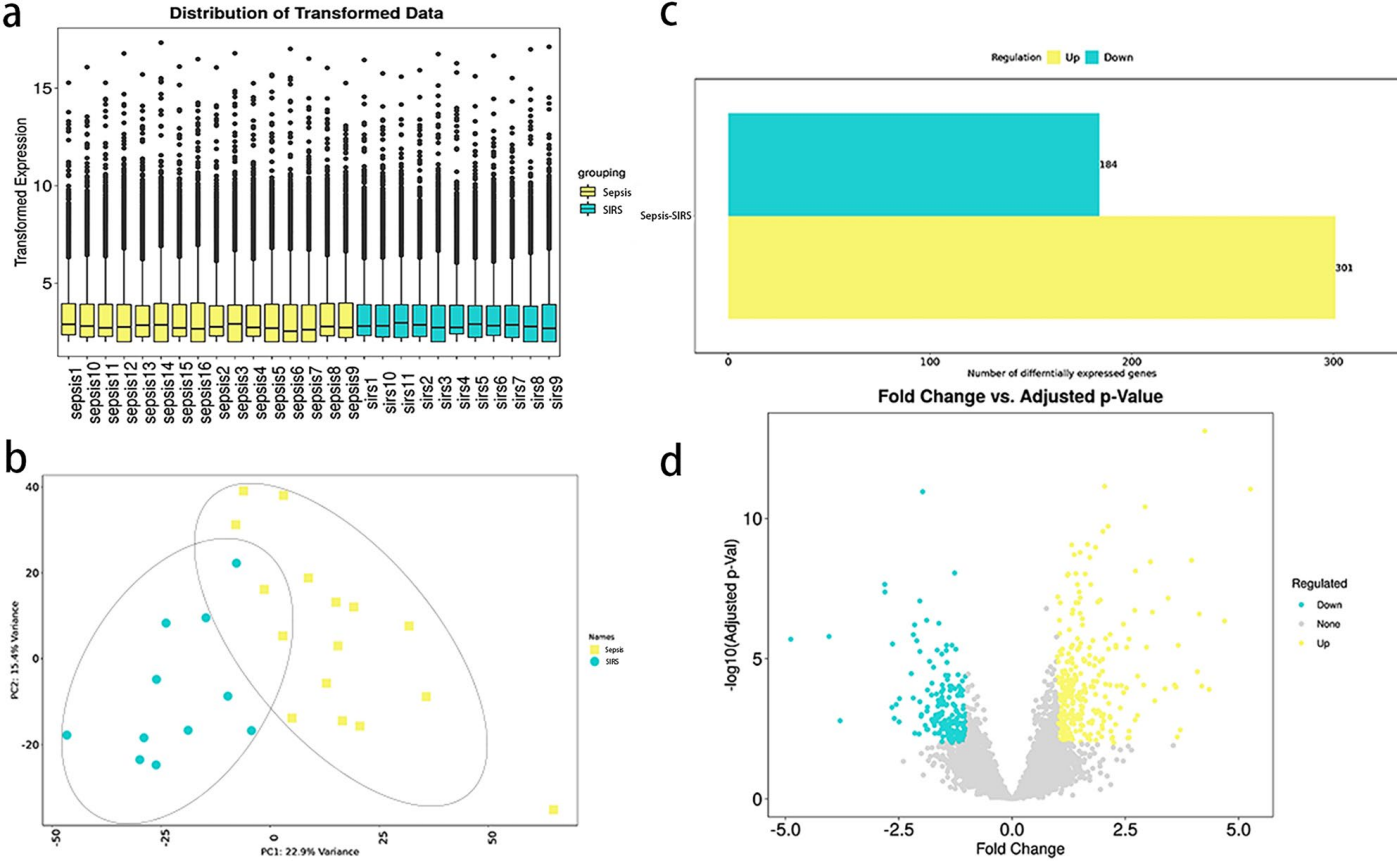
Genomic data quality control and differential screening. (**a**) The y-axis of the box plot represents the logarithmised FPKM (number of fragments per thousand exon patterns per million mapped fragments), also known as log10(FPKM), which indicates that the data for each sample are homogenised, distributed at the same level and comparable; (**b**) principal component analysis shows that the two groups can be clearly differentiated and have no outliers; (**c**) the bar graphs show that the differential analysis screened out up-regulated (yellow) 184 and down-regulated (green) 301 genes; (**d**) volcano plot showing the up-regulated (yellow) and down-regulated (green) genes screened by differential analysis, with horizontal coordinates for gene expression in the sepsis group and vertical coordinates for gene expression in the SIRS group.

Box plots and principal component analysis of the mRNAs obtained by sequencing showed good homogeneity and intergroup differentiation between samples from the SIRS and sepsis groups, with no abnormal samples (Fig. 1a and b).The data from the two groups were analyzed using analysis of variance, with a threshold of |FC|≥ 2.0 and FDR < 0.05, resulting in the identification of 485 genes that were differentially expressed (Fig. 1c), of which 301 genes were up-regulated for expression in yellow, 184 genes were down-regulated for expression in blue, and grey was for no differentially expressed genes (Fig. 1d).

### Non-targeted metabolomics analysis

#### Classification of metabolites

The obtained specimens were thawed slowly at 4 °C, the extraction solution was added and centrifuged several times, and the supernatant was subjected to LC–MS/MS technology for metabolite separation and detection to separate and detect the metabolites, and the raw data of metabolites were obtained after passing the on-board quality control and the data processing (mainly including peak extraction, intra- and inter-group retention time correction, addition and combination of ions, missing value filling, background peak labelling and data quality control) was carried out by Compound Discoverer 3.1 (Thermo The data were processed by Compound Discoverer 3.1 (Thermo Fisher Scientific, USA) (mainly including peak extraction, intra- and inter-group retention time correction, addition ion merging, missing value filling, background peak labelling, and data quality control), and then the molecular weights, retention times, peak areas, and identifications were combined with those of KEGG (Kyoto Encyclopedia of Genes and Genetics), BGI The molecular weights, retention times, peak areas and identification results were then combined with the KEGG (Kyoto Gene and Kyoto Encyclopedia), BGI Library, Chemspider, Lipidmaps and HMDB databases for metabolite identification, taxonomic annotation and pathway annotation.

#### Screening for differential metabolites

**Figure 2:**
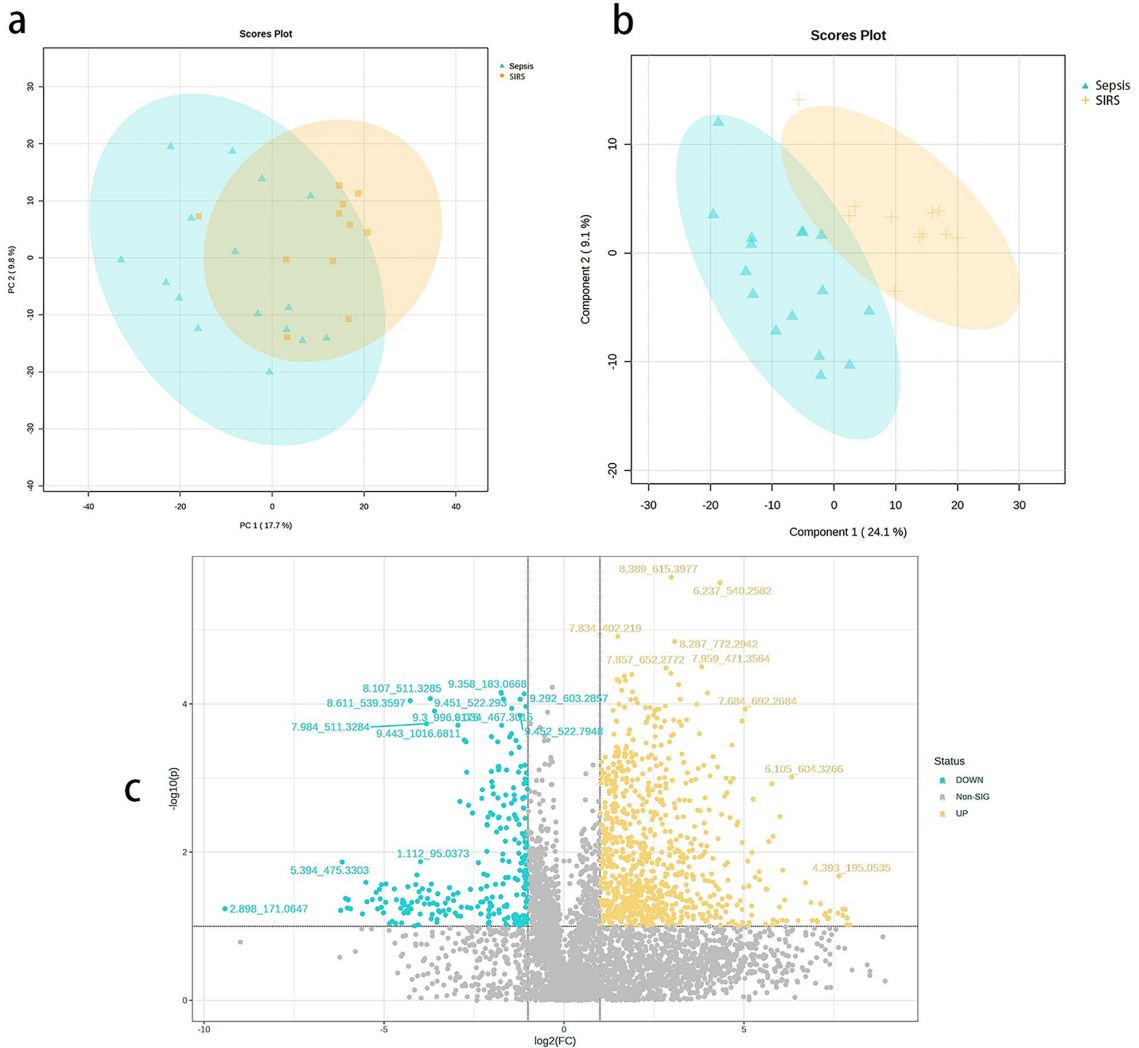
Metabolomic data allegation and difference screening. (**a**) Principal component analysis showed that the two groups were clearly distinguishable with no outliers; (**b**) OPLS-DA analysis showed a clear separation between sepsis patients and SIRS subjects, indicating significant differences in their serum non-targeted metabolomics profiles; (**c**) volcano plots showed that the differential analysis screened for up-regulated (yellow) 831 and down-regulated (green) 1083 metabolites, with the horizontal coordinates being the sepsis group's gene expression and vertical coordinates for the SIRS group.

Following the analysis of non-targeted metabolomics and quality control of peripheral blood from 16 patients with sepsis and 11 patients with SIRS using LC–MS/MS technology, principal component analysis revealed a high level of uniformity and distinctiveness between samples from the sepsis and SIRS groups, without any exceptional samples (Fig. 2a). The OPLS-DA analysis revealed a clear demarcation between sepsis patients and SIRS subjects, indicating significant differences in their serum untargeted metabolomic profiles (Fig. 2b). A grand total of 7140 active substances were acquired, comprising 5077 in the mode of positive ions and 2063 in the mode of negative ions. By applying a threshold of |Fc|≥ 2.0 and P < 0.05, a total of 1083 compounds showing differential expression were identified. Among these, 831 metabolites were up-regulated and represented in red, while 252 metabolites were down-regulated and represented in blue (Fig. 2c).

### Combined metabolomics and transcriptomics analyses

**Figure 3:**
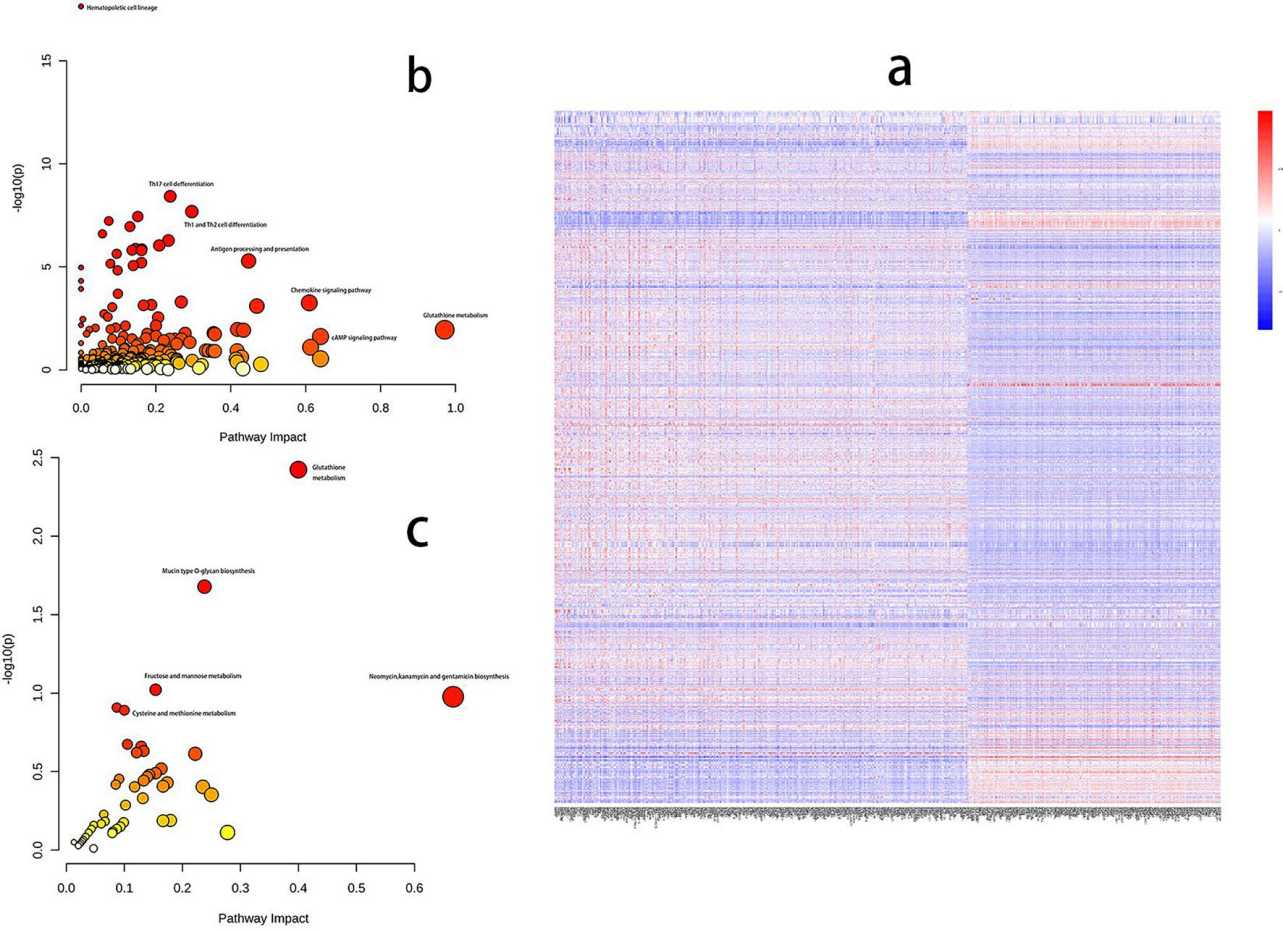
Intergroup analysis (**a**) intergroup correlation heatmap showing that sepsis patients differ significantly from SIRS subjects in terms of genomic and non-targeted metabolomic data; genes characterised based on metabolic pathway (**b**) co-regulation are mainly involved in haematopoietic cell profiles, Th17 cell differentiation, Th1 and Th2 cell differentiation, cellular adhesion molecules, Staphylococcus aureus infection, graft-versus-host disease (**b**) metabolites are mostly enriched in glutathione metabolism, mucin-type *O*-glycan biosynthesis, fructose and mannose metabolism, and cysteine and methionine metabolism. It has an important role in energy production and human immunity.

To build a thorough sepsis profile and establish the correlation between metabolites and genes, multi-omics analyses were conducted by integrating transcriptomics and non-targeted metabolic data from identical biological samples. Metabolomics and transcriptomics intergroup correlation analyses were performed through the omicshare online platform, and intergroup correlation heatmaps revealed significant differences between sepsis patients and SIRS subjects in terms of genomic and non-targeted metabolomics data (Fig. 3a). The potential components of the two histological datasets were highly correlated, indicating good intergroup correlation between the two datasets. Significant positive and negative correlations were found between genes and metabolites. Identified were clusters of co-regulatory characteristics closely linked to potential elements of the multi-omics dataset, possibly serving as features of sepsis. MetaboAnalyst 5.0 online software was used to perform multi-omics analyses, which integrated transcriptomics and non-targeted metabolic data from the same biological samples within the framework of metabolic pathways, with the aim of identifying relationships between metabolites and genes. The functional enrichment analyses indicated that genes exhibiting co-regulatory patterns were primarily associated with biological processes including profiling of haematopoietic cells, differentiation of Th17 cells, differentiation of Th1 and Th2 cells, molecules involved in cell adhesion, infections caused by Staphylococcus aureus, graft-versus-host disease, the immune network in the intestines used for IgA production, and processing and refinement of antigens (Fig. 3b), with a total of 40 differentially screened genes (CD8A, ITGB2, VCAN, ITGA4, CD2, ITGAM, HLA-DMA, CD86, CD28, ICOS, ITGA6, CD59, CR1, CD44, CD36, CSF3R, IL1R1, IL4R, CD1A, IL7R, CFD, FCAR, C3AR1, ITGAL, NFKBIA, LCK, IL2RG, RORA, RUNX1, CD3E, CD3G, CD3D, CD247, JAK1, KIR2DL1, CD80, ITGA4, CD74, KIR2DS2, HSPA1A).Glutathione metabolism, mucin-type *O*-glycan biosynthesis, fructose and mannose metabolism, and cysteine and methionine metabolism are the main pathways where metabolites are predominantly concentrated. Have important roles in energy production and human immunity (Fig. 3c).The findings indicate possible interaction between the regulation of inflammation, immune modulation, and amino acid metabolism, offering a potential strategy to mitigate the systemic inflammatory response syndrome in sepsis.

### Meta-analysis

**Figure 4:**
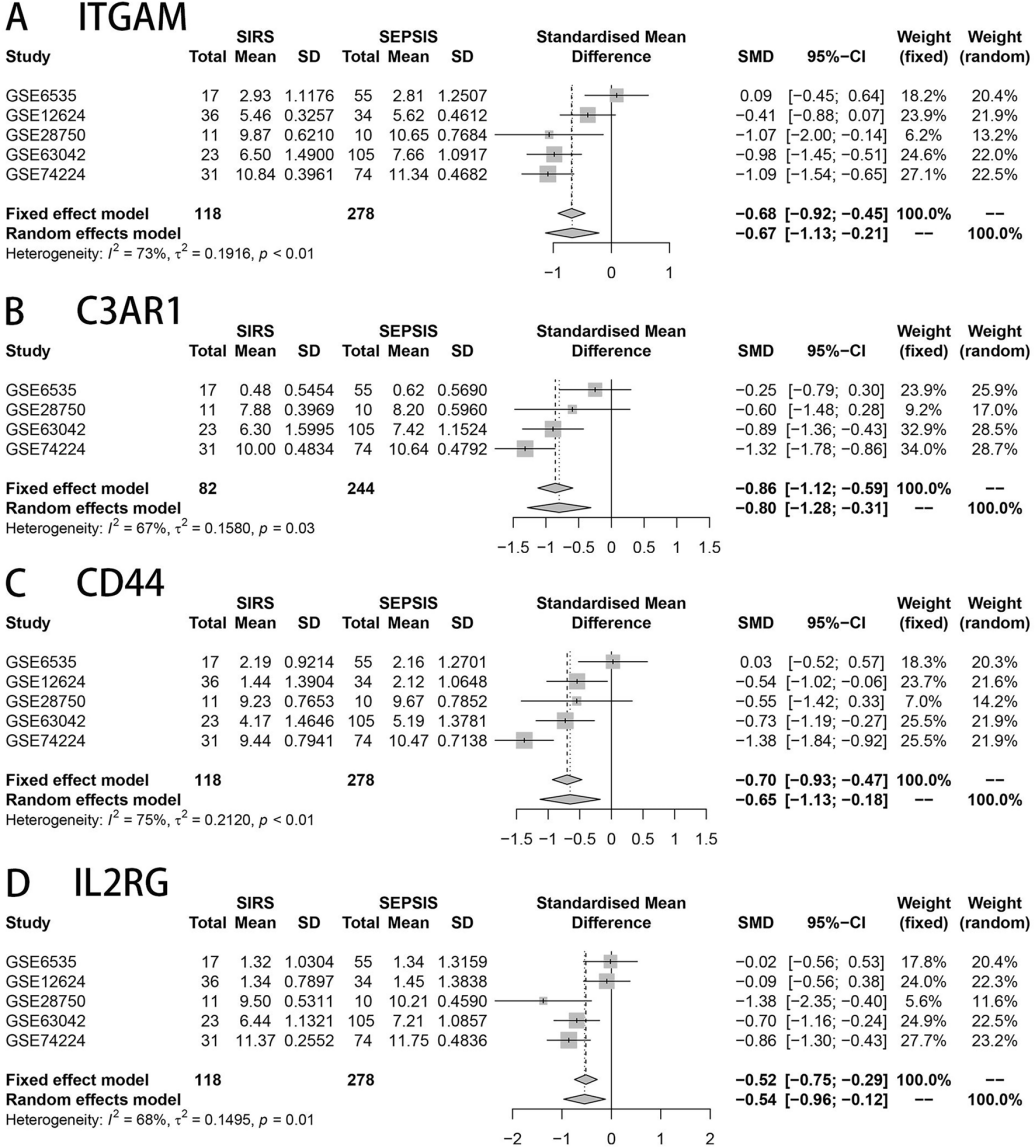
(**A**–**D**) represents ITGAM, CD44, C3AR1, IL2RG genes in GSE6535, GSE12624, GSE28750, GSE63042, GSE74224 of SIRS and sepsis groups respectively, the four core genes were lowly expressed in the normal group, and highly expressed in the sepsis group, the difference was statistically significant (*P* < 0.05).

The analysis of more than 40 genes that differ across transcriptional levels was conducted using the sepsis database in the GEO public database (GSE6535, GSE12624, GSE28750, GSE63042, GSE74224). The results indicated that ITGAM, CD44, C3AR1, and IL2RG exhibited significantly higher expression in the sepsis group compared to the SIRS group. This difference was found to be statistically significant (*P* < 0.05) as shown in Fig. 4A–D.

### Single-cell RNA sequencing

**Figure 5:**
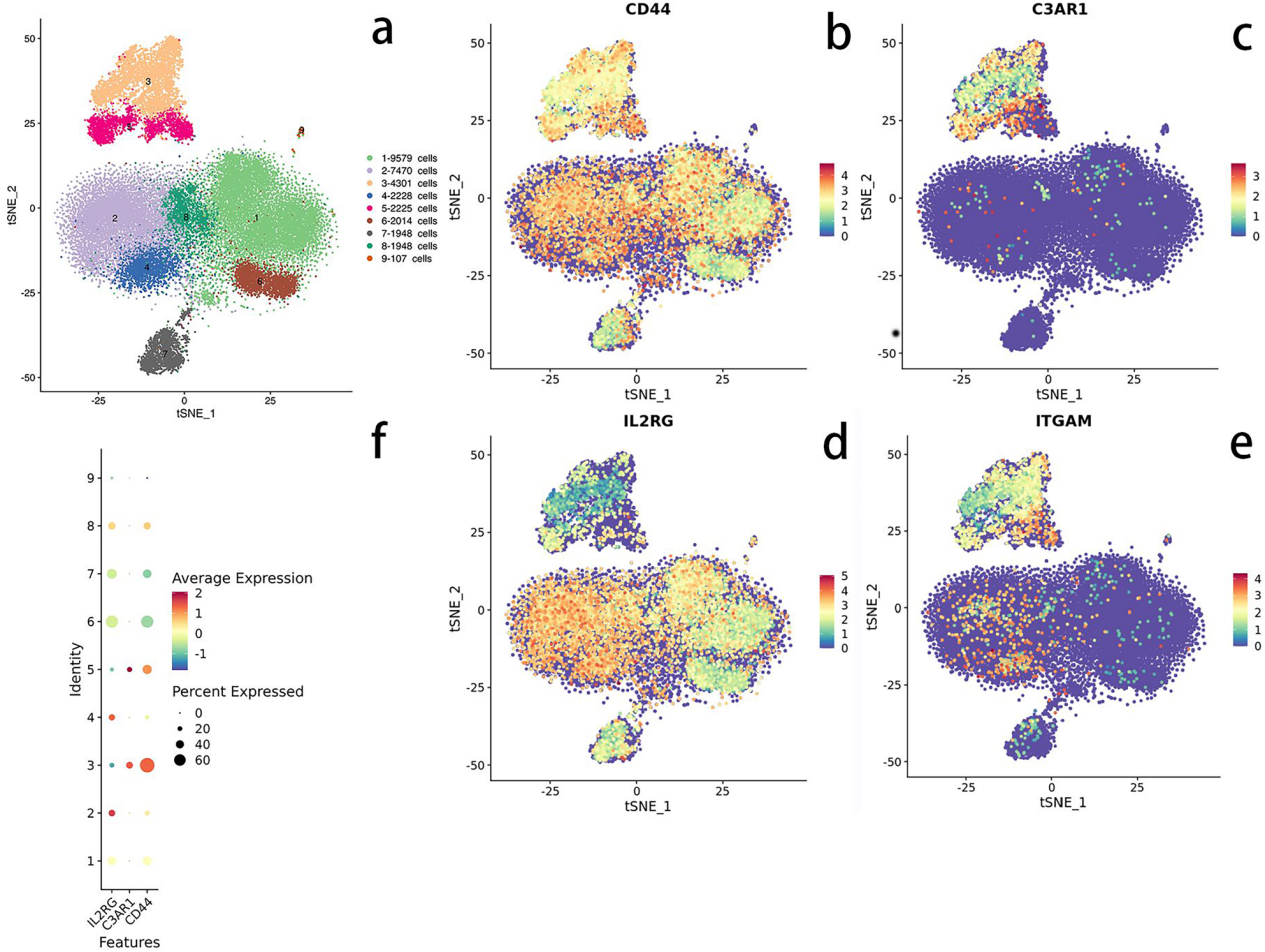
Single-cell RNA sequencing. (**a**) General diagram of mixed sample sequencing. Cell populations 1, 2, 6 and 8 are T cells, 3 and 5 are macrophages, 4 are NK cells, 7 are B cells and 9 are platelets. (**c**, **e**) Suggests that the core genes ITGAM and C3AR1 are mainly localised in cell populations 3 and 5, i.e. the macrophage lineage. (**b**, **d**) The core genes CD44 and IL2RG are widely localised to various cell populations and are expressed to varying degrees in all types of cell populations.

The quantity of excellent cells per specimen ranged from 4000 to 10,000, while the resulting quantity of cells after removing duplicate cells, multiple cells, and apoptotic cells ranged from 3108 to 8509. The mean count of distinct molecular identifiers (UMIs) per cell varied from 519 to 8529, and the mean count of genes per cell ranged from 343 to 2337. Following the process of descending clustering, the cells were categorized into 9 distinct clusters. The identified cell types, determined by marker genes, included B cells, NK cells, T cells, platelets, and monocytes. Figure showed that platelets were represented by macrophages 3 and 5, NK cells 4, T cells 1, 2, 6, and 8, as well as B cells 7 and 9(Fig. 5A). ITGAM and C3AR1 were mainly localized in 3 and 5 cell clusters, i.e., macrophage lineage (Fig. 5C,E); CD44 and IL2RG were expressed to varying degrees in all types of cell clusters (Fig. 5B,D).

## Discussion

Sepsis is a syndrome of organ dysfunction that endangers life and is triggered by an uncontrolled reaction of the body to infection^22^. SIRS is a systemic inflammatory response due to infectious or non-infectious factors. The systemic inflammatory response is not caused by a single factor but is a complex pathological syndrome involving a large number of systemic responses. Inflammatory mechanisms involve humoral and cellular responses, complement, and cytokine cascades. Sepsis is also not just a systemic inflammatory response; there is also an anti-inflammatory response and adaptive changes to inflammation in the organism. In addition to inducing inflammation and an anti-inflammatory response, infection can cause abnormalities in coagulation-anticoagulation, neuro-endocrinology, and metabolism.The challenge in diagnosing and treating sepsis stems from the lack of complete understanding of its pathomechanisms and the absence of knowledge about the primary targets linked to prognosis, making precise targeted therapy impossible. Therefore breakthroughs in the pathophysiological mechanisms of SIRS and sepsis are needed for better differential diagnosis and treatment. Metabolic changes have been discovered to be linked with sepsis^23^.Elucidating the pathophysiological features of sepsis could help reduce its high morbidity and mortality. In this study, we used RNA sequencing technology and combined it with untargeted metabolomics analysis to reveal the alteration of genes to metabolites in sepsis, screening 40 potential core genes. The kegg enrichment analysis focused on the core genes and revealed significant enrichment of biological processes associated with the regulation of the human immune system, metabolism of amino acids, and the inflammatory response. Through meta-analysis, four potential key genes, namely ITGAM, CD44, C3AR1, and IL2RG, were identified. These genes exhibited similar expression patterns to their corresponding metabolites, indicating concurrent alterations at both transcriptional and metabolic levels. Further investigation is warranted for two crucial genes that were predominantly localized in macrophages and exhibited high expression in the sepsis group, indicating their potential as valuable leads for sepsis treatment.

Existing reports indicate that ITGAM (alpha M integrin subunit) plays a crucial role in the activation and movement of inflammatory cells^24^.Earlier investigations demonstrated that ITGAM primarily facilitates the advancement of sepsis by enhancing the liberation of HMGB1 through nuclear and cytoplasmic translocation and activation. Antibodies or inhibitors that block ITGAM protect mice from the mortality linked to LPS and bacterial sepsis^25^.The current investigation shows that ITGAM is primarily found in macrophages and is significantly expressed in individuals with sepsis^26^. Additionally, the integrin CD11b, encoded by ITGAM, is present on the surface of macrophages and plays a role in adhesion, migration, and cell-mediated cytotoxicity. Knocking down ITGAM resulted in CD11b deficiency, which increased the pro-inflammatory activity of macrophages. This led to higher production of TNF-α and IL-6, resulting in increased susceptibility to sepsis induced by methicillin-resistant Staphylococcus aureus (MRSA) infection. Additionally, CD11b deficiency upregulated the expression of IL-4-induced anti-inflammatory mediators. Macrophages inhibit T cell activation through the production of IL-10 and arginase-1. Low expression of ITGAM enhances NF-κB signalling activation and Akt phosphorylation, promoting the functional activation of macrophages with pro-inflammatory and immunomodulatory phenotypes. Additionally, a different research discovered that ITGAM also has a significant impact on sepsis caused by methicillin-resistant Staphylococcus aureus (MRSA).After being infected with MRSA, the mortality rate of ITGAM knockout mice was considerably greater compared to that of control mice^26^.

It was previously believed that C3AR1 (receptor 1 for complement component 3a) was limited to the innate immune response and had a function in the complement cascade. However, the involvement of C3AR1 has been expanded to encompass cancer, neurogenesis, and pituitary hormone release^27^.Our research findings indicate a significant expression of C3AR1 in individuals afflicted with sepsis, predominantly within macrophages. In sepsis, heightened C3AR1 expression triggers augmented MAPK signaling downstream of TLR4 in macrophages and Ifnar stimulation, thereby amplifying the production of pro-inflammatory mediators.Additionally, C3AR1 might play a role in the differentiation between M1 and M2 macrophages^28^. This differentiation leads to a shift towards an M2 phenotype, where macrophages can become excessively activated and produce an abundance of pro-inflammatory cytokines in the early stages. If the pro-inflammatory response induced by macrophages cannot be properly controlled, it may result in a cytokine storm, leading to macrophage apoptosis and ultimately causing immunosuppression^29–32^.Thus,C3AR1 may contribute significantly to the pathophysiology of sepsis. The identification of the central gene may contribute to prognostic markers or therapeutic targets in sepsis.

For this research, we employed RNA sequencing of human peripheral blood, liquid chromatography coupled with mass spectrometry (LC–MS/MS technology), and 10× single-cell RNA sequencing. Additionally, we utilized data from the GEO public database to identify the key genes associated with sepsis clinical phenotypes. Our aim was to investigate the expression of these core genes from various perspectives, offering valuable insights for future comprehensive investigations. The main focus of this research was to examine the coherence between gene and metabolite expression patterns in blood. Specifically, our focus was primarily on the augmented manifestation of elemental components in plasma, which may potentially be linked to intercellular communication or leakage following cellular death. However, a considerable number of crucial genes remain unexpressed in plasma, specifically certain transcription factors that undergo relocation to the nucleus. One limitation of this research is its observational nature, lacking any supplementary functional validation of the target genes.

## Conclusion

Macrophages predominantly express the key genes ITGAM and C3AR1, which play a significant role in inflammatory and immune responses. Moreover, these genes exhibit high expression in the plasma of sepsis patients, indicating their potential as valuable research targets for sepsis.

### Supplementary Information

Supplementary Figure 1.

Supplementary Legend.

Supplementary Information.

## Data Availability

The data that support the findings of this study are available from the corresponding author, [Muhu Chen], upon reasonable request.
