# Supplementary figures and images for: Integrative analysis of metabolomics and transcriptomics to uncover biomarkers in sepsis
Source: Sci Rep. 2024 Apr 27;14:9676. doi: 10.1038/s41598-024-59400-0 (PMC11055861; doi:10.1038/s41598-024-59400-0)

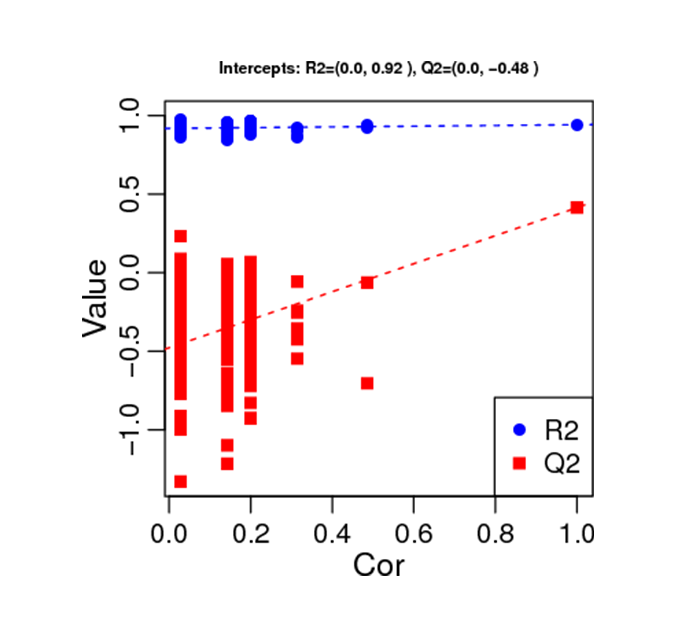

Supplement: Supplementary file 1 — Supplementary Figure 1. [file 41598_2024_59400_MOESM1_ESM.tif]
