# Supplementary material for: Integrative analysis of metabolomics and transcriptomics to uncover biomarkers in sepsis
Source: Sci Rep. 2024 Apr 27;14:9676. doi: 10.1038/s41598-024-59400-0 (PMC11055861; doi:10.1038/s41598-024-59400-0)
Supplement: Supplementary file 2 — Supplementary Legend. [file 41598_2024_59400_MOESM2_ESM.docx]

Supplementary images: The two rightmost points in the figure are the actual R2Y and Q2 values of the model, and the remaining points are the R2Y and Q2 values obtained by randomly arranging the samples used. This result is mainly used to judge whether the model is overfit and the validity of the model.
