# Supplementary material for: Integrative analysis of metabolomics and transcriptomics to uncover biomarkers in sepsis
Source: Sci Rep. 2024 Apr 27;14:9676. doi: 10.1038/s41598-024-59400-0 (PMC11055861; doi:10.1038/s41598-024-59400-0)
Supplement: Supplementary file 3 — Supplementary Information. [file 41598_2024_59400_MOESM3_ESM.docx]

**1 Untargeted metabolomics analysis process**

The analysis process of untargeted metabolomics is divided into two parts: experimental and bioinformatics analysis. The experimental procedure includes metabolite extraction, LC-MS/MS detection, etc. Bioinformatics analysis mainly includes: data preprocessing, data quality control, statistical analysis, screening for differential metabolites, pathway annotation and pathway enrichment analysis.


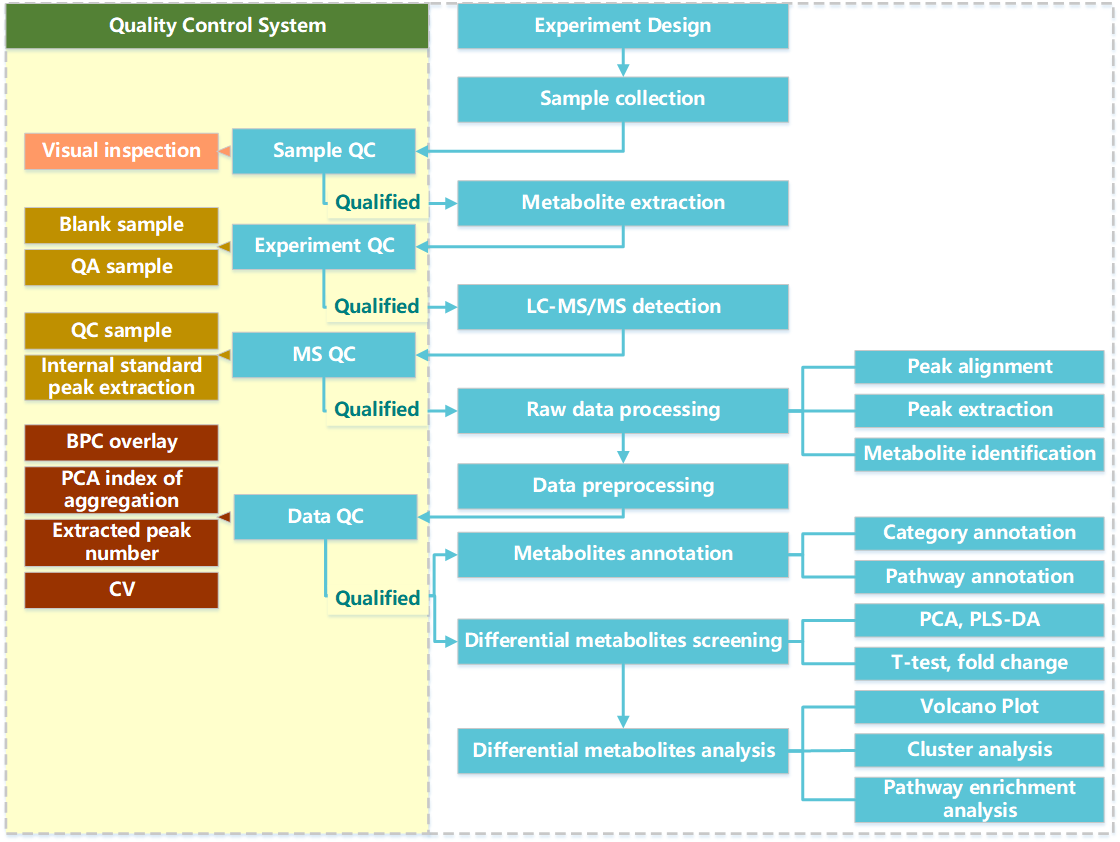


**Figure 1  Untargeted metabolomics flow chart.**

**2 Experimental method**

After receiving the sample, the sample center renumbered each sample to better manage and store the sample, which was used during the experimental process.

**2.1 Main instruments and reagents**

Ultra Performance Liquid Chromatography:(Waters 2D UPLC, Waters, USA)

High Resolution mass spectrometer:(Q Exactive, Thermo Fisher Scientific, USA)

Chromatographic Column:ACQUITY UPLC BEH C18 (1.7 μm,2.1*100 mm,Waters, USA)

Low Temperature High Speed Centrifuge:(Centrifuge 5430,Eppendorf)

Vortex:(QL-901, Kylin-bell Lab Instruments Co., Ltd.,China)

Ultrapure Water Systems:(Milli-Q Integral,Millipore Corporation,USA)

Vacuum Concentrator:（Maxi Vacbeta,GENE COMPANY）

The internal standard mix (IS) contains: L-Leucine-d3,L-PHENYLALANINE (13C9, 99%),L-Tryptophan-d5,Progesterone-2,3,4-13C3.

MS-grade methanol (A454-4) and acetonitrile (A996-4) were purchased from Thermo Fisher Scientific (USA). Formic acid was purchased from DIMKA (50144-50ml, USA) and ammonium formate (17843-250G,Honeywell Fluka, USA) was obtained from Fluka. Ultrapure water was filtered through the Milli-Q system.

**2.2 Metabolite extraction**

Metabolite extraction was primarily performed according to previously reported methods[^[6]^](https://www.nature.com/articles/nprot.2011.335)[^[7]^](https://pubmed.ncbi.nlm.nih.gov/24820162/). In short, 100 µL samples were extracted by directly adding 300 µL of precooled methanol and acetonitrile (2:1, v/v), internal standards mix 1 (IS1) and internal standards mix 2 (IS2) were added for quality control of sample preparation. After Vortex for 1min and incubate at -20 °C for 2 hours, samples were centrifuged for 20 min at 4000 rpm, and the supernatant was then transferred for vacuum freeze drying. The metabolites were resuspended in 150 µL of 50% methanol and centrifuged for 30 min at 4000 rpm, and the supernatants were transferred to autosampler vials for LC-MS analysis. A quality control (QC) sample was prepared by pooling the same volume of each sample to evaluate the reproducibility of the whole LC-MS analysis.

**2.3 LC-MS/MS Analysis**

This experiment used a Waters 2D UPLC (waters, USA) tandem Q Exactive high resolution mass spectrometer (Thermo Fisher Scientific, USA) for separation and detection of metabolites.

1.Chromatographic conditions

The samples were analyzed on a Waters 2D UPLC (Waters, USA), coupled to a Q-Exactive mass spectrometer (Thermo Fisher Scientific, USA) with a heated electrospray ionization (HESI) source and controlled by the Xcalibur 2.3 software program (Thermo Fisher Scientific, Waltham, MA, USA). Chromatographic separation was performed on a Waters ACQUITY UPLC BEH C18 column (1.7 μm, 2.1 mm × 100 mm, Waters, USA), and the column temperature was maintained at 45 °C. The mobile phase consisted of 0.1% formic acid (A) and acetonitrile (B) in the positive mode, and in the negative mode, the mobile phase consisted of 10 mM ammonium formate (A) and acetonitrile (B). The gradient conditions were as follows: 0-1 min, 2% B; 1-9 min, 2%-98% B; 9-12 min, 98% B; 12-12.1 min, 98% B to 2% B; and 12.1-15min, 2% B. The flow rate was 0.35 mL/min and the injection volume was 5 μL.

2. Mass spectrometry conditions

The mass spectrometric settings for positive/negative ionization modes were as follows: spray voltage, 3.8/−3.2 kV; sheath gas flow rate, 40 arbitrary units (arb); aux gas flow rate, 10 arb; aux gas heater temperature, 350 °C; capillary temperature, 320 °C. The full scan range was 70–1050 m/z with a resolution of 70000, and the automatic gain control (AGC) target for MS acquisitions was set to 3e6 with a maximum ion injection time of 100 ms. Top 3 precursors were selected for subsequent MSMS fragmentation with a maximum ion injection time of 50 ms and resolution of 17500, the AGC was 1e5. The stepped normalized collision energy was set to 20, 40 and 60 eV.

In order to provide more reliable experimental results during instrument testing, the samples are randomly ordered to reduce system errors. A QC sample is interspersed for every 10 samples.

**3 Databse introduction**

**3.1 BGI Library**

BGI Library is a inhouse-developed standard database, including retention time (RT), MS1 spectrum (MS1), and MS2 spectrum (MS2) of all standards. Key primary metabolites and metabolic intermediates in the key metabolic pathways are covered, including carboxylic acids, amino acids, biogenic amines, polyamines, nucleotides, coenzymes and vitamins, monosaccharides and disaccharides, fatty acids, lipids, steroids and hormones .

**3.2 mzCloud database**

[mzCloud](https://www.mzcloud.org/) is Thermo's high-resolution tandem mass spectrometry cloud database. It contains a high-resolution and accurate MSn spectrum library of tens of thousands of compounds. Currently, the number of spectra exceeds 2.8 million, and the chemical structure information exceeds 1.06 million. It is still being updated. All data is measured by standard products and is subject to strict manual corrections. It is the most comprehensive database.

**3.3 Chemspider Database**

[Chemspider](http://www.chemspider.com/) is an online chemical database that provides up to millions of chemical structures. There are 354 optional data sources. As a plug-in for the Compound Discoverer software, the database selected for compound identification is HMDB, KEGG, LipidMaps, etc.

**3.4 HMDB database**

The [HMDB](http://www.hmdb.ca/) database is currently one of the most comprehensive databases of human metabolites. The data in the database covers the following three categories: a. chemical data, b. clinical data, c. analytical biology and biochemical data. Both water soluble and fat soluble metabolites are included. In addition, the included information also provides links to other databases (eg KEGG, PubChem, etc.) for easy access.

**3.5 KEGG database**

The [KEGG](http://www.kegg.jp/) database is designed to understand the functions and interactions of genes, proteins, and metabolites in biological systems such as cells, tissues, and so on. Information on metabolite-related metabolic pathways, human diseases, and drug discovery can be found. Metabolites and metabolic pathways in this database cover two broad categories: eukaryotes (animals, plants, fungi, and protists) and prokaryotes.

**3.6 Lipidmaps database**

[Lipidmaps](http://www.lipidmaps.org/) is a database of lipids. It has collected more than 40,000 kinds of lipids and classified the lipids into 8 categories: fatty acids. Classes, glycerides, glycerophospholipids, sphingolipids, sterols, pregnenol ketones, glycolipids and polypolyethylenes. The LipidMaps database is generally preferred for lipid identification.

**Data preprocessing and quality control**

**1.1 Data Preprocessing**

The mass spectrometry raw data (raw file) collected by LC-MS/MS was imported into Compound Discoverer 3.1 (Thermo Fisher Scientific, USA) for data processing, including: peak extraction, retention time correction within and between groups, additive ion pooling. , missing value filling, background peak labeling, and metabolite identification, and finally information on compound molecular weight, retention time, peak area, and identification results were exported. The identification of metabolites is a combined result of BGI Library (BGI inhouse-developed standard library), mzCloud and ChemSpider (HMDB, KEGG, LipidMaps) databases.

The results of the Compound Discoverer 3.1 export are imported into metaX for data preprocessing, including: 1. Normalize the data using the Probabilistic Quotient Normalization (PQN[^[2]^](https://www.ncbi.nlm.nih.gov/pmc/articles/PMC4831991/)) to obtain the relative peak area. 2. Correct the batch effect using QC-RLSC(Quality control-based robust LOESS signal correction); 3. Calculate the CV (Coefficient of Variation) of the relative peak area in all QC samples , and delete the compounds with CV greater than 30%.

Probabilistic Quotient Normalization (PQN): Probabilistic quotient normalization is the calculation of the average of all QC samples for each feature to obtain a reference vector; calculating the median between the reference vector and each sample and obtaining the coefficient vector associated with each sample, then normalization is performed by dividing each sample by the median of the coefficient vectors (the median of each sample is different).

QC-RLSC: Local polynomial regression fitting signal correction (QC-RLSC) based on QC sample information is a more effective data correction method in metabolomics area data analysis.

**1.2 Data Quality Control**

Data quality was assessed by repeatability of QC sample detection. The content includes chromatogram overlap of the QC sample, PCA, peak number and peak response intensity difference.

**(1). Chromatogram overlap of QC samples**

The BPC (base peak chromatogram) of all QC samples were overlapped, the spectrum overlap was good, and the retention time and peak response intensity fluctuated little, indicating that the instrument was in good condition and the signal was stable during the whole sample detection and analysis.


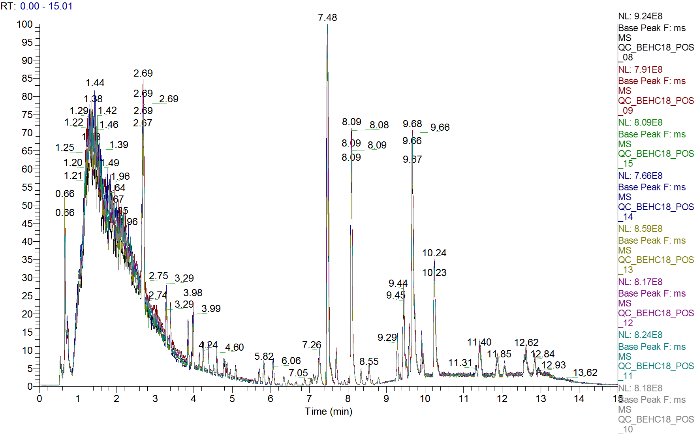

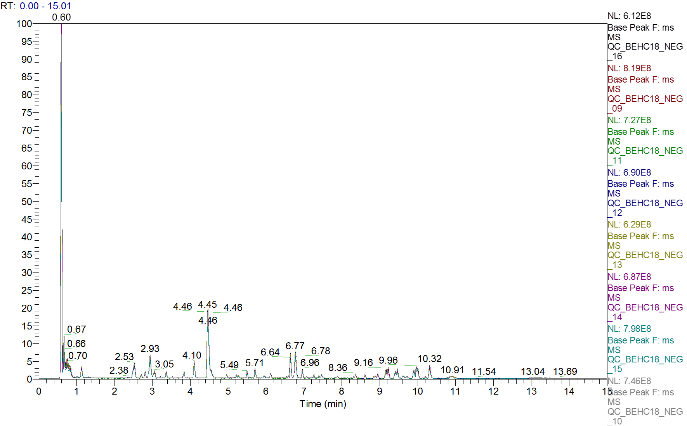


**BPC overlay of QC samples.**

BPC is a map that continuously depicts the intensity of the strongest ions in the mass spectrum at each time point.

**(2). Principal Component Analysis (PCA) for all samples**


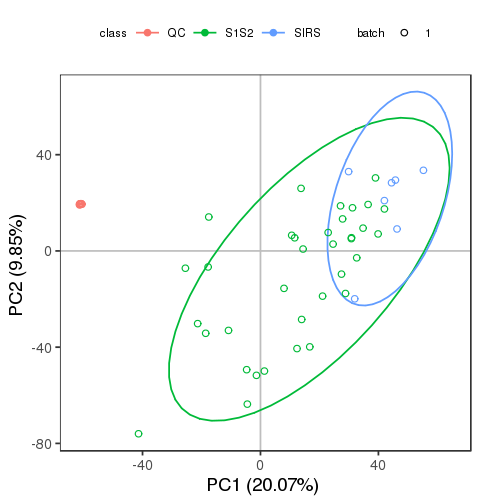


**PCA score chart for all samples.**

The abscissa is the first principal component PC1, the ordinate is the second principal component PC2, and the ellipse in the PCA score graph is 95% confidence interval. Each dot represents a sample, and different groups are labeled with different colors. The number is the score of the principal component, which represents the percentage of the explanation on overall variance of the specific pricipal component.

**2.1 Classification and functional annotation of metabolites**

Refer to the KEGG and HMDB databases to classify and annotate the identified metabolites to understand the classification of the metabolites. The statistical chart for the number of each classification of metabolite is shown in the following figures. Others means that classification information is the remaining categories, and the evaluation results without classification information do not participate in statistics.


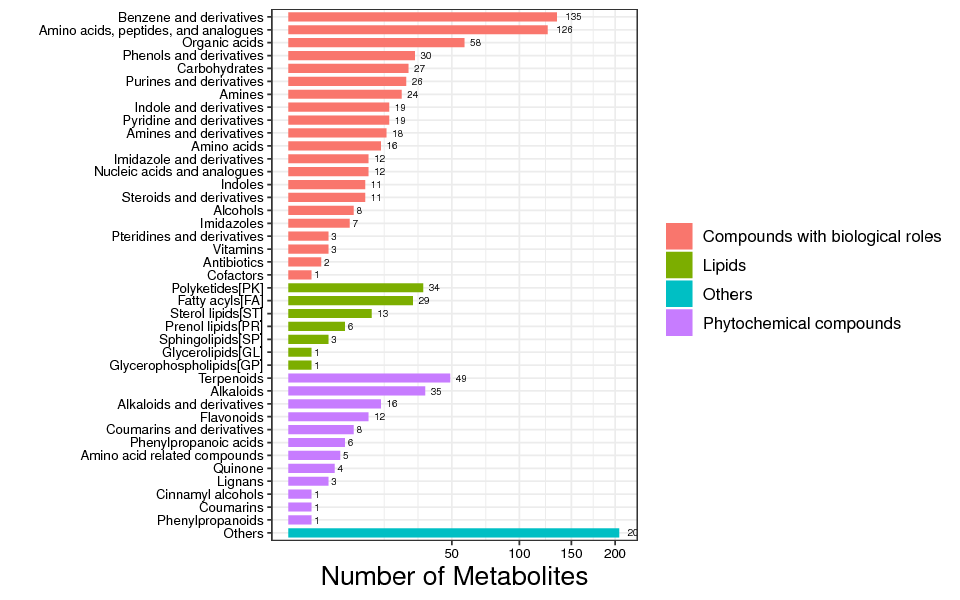


**Metabolite classification bar chart**

The X axis represents the number of metabolite classifications, and the Y axis represents the metabolite classification entries.

The KEGG database was used to annotate the identified metabolites to understand the functional characteristics of different metabolites and determine the main biochemical metabolic pathways and signal transduction pathways involved in the metabolites. The number of metabolites in each type of metabolic pathway is shown in the following figures.


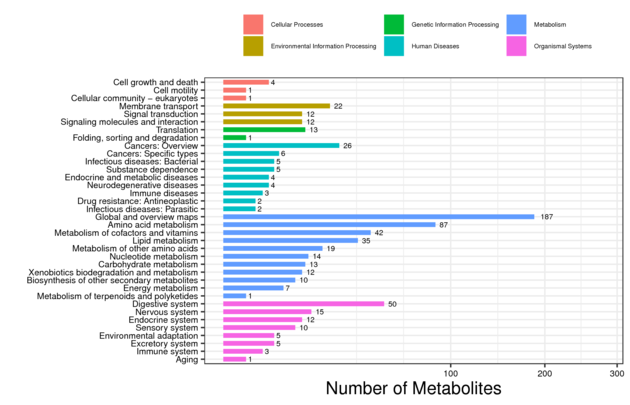


**KEGG function comment bar chart.**

X axis represents the number of metabolite annotations, and the Y axis represents the annotated KEGG Pathway.
